# Heterologous production of rhamnolipids in *Pseudomonas chlororaphis* subsp chlororaphis ATCC 9446 based on the endogenous production of *N*‐acyl‐homoserine lactones

**DOI:** 10.1111/1751-7915.14377

**Published:** 2023-12-02

**Authors:** Abigail González‐Valdez, Adelfo Escalante, Gloria Soberón‐Chávez

**Affiliations:** ^1^ Departamento de Biología Molecular y Biotecnología, Instituto de Investigaciones Biomédicas Universidad Nacional Autónoma de México Coyoacan Mexico; ^2^ Departamento de Ingeniería Celular y Biocatálisis, Instituto de Biotecnología Universidad Nacional Autónoma de México Cuernavaca Mexico

## Abstract

Rhamnolipids (RL) are biosurfactants naturally produced by the opportunistic pathogen *Pseudomonas aeruginosa*. Currently, RL are commercialized for various applications and produced by *Pseudomonas putida* due to the health risks associated with their large‐scale production by *P. aeruginosa*. In this work, we show that RL containing one or two rhamnose moieties (mono‐RL or di‐RL, respectively) can be produced by the innocuous soil‐bacterium *Pseudomonas chlororaphis* subsp chlororaphis ATCC 9446 at titres up to 66 mg/L (about 86% of the production of *P. aeruginosa* PAO1 in the same culture conditions). The production of RL depends on the expression of *P. aeruginosa* PAO1 genes encoding the enzymes RhlA, RhlB and RhlC. These genes were introduced in a plasmid, together with a transcriptional regulator (*rhlR*) forming part of the same operon, with and without RhlC. We show that the activation of *rhlAB* by RhlR depends on its interaction with *P. chlororaphis* endogenous acyl‐homoserine lactones, which are synthetized by either PhzI or CsaI autoinducer synthases (producing 3‐hydroxy‐hexanoyl homoserine lactone, 3OH‐C6‐HSL, or 3‐oxo‐hexanoyl homoserine lactone, 3O‐C6‐HSL, respectively). *P. chlororaphis* transcriptional regulator couple with 3OH‐C6‐HSL is the primary activator of gene expression for phenazine‐1‐carboxylic acid (PCA) and phenazine‐1‐carboxamide (PCN) production in this soil bacterium. We show that RhlR coupled with 3OH‐C6‐HSL or 3O‐C6‐HSL promotes RL production and increases the production of PCA in *P. chlororaphis*. However, PhzR/3OH‐C6‐HSL or CsaR/3O‐C6‐HSL cannot activate the expression of the *rhlAB* operon to produce mono‐RL. These results reveal a complex regulatory interaction between RhlR and *P. chlororaphis* quorum‐sensing signals and highlight the biotechnology potential of *P. chlororaphis* ATCC 9446 expressing *P. aeruginosa rhlAB‐R* or *rhlAB‐R‐C* for the industrial production of RL.

## Graphical abstract

The heterologous production of the biosurfactant rhamnolipids in *Pseudomonas chlororaphis* ATCC 9446 was attained by the expression of the *P. aeruginosa* genes coding for the RhlA, RhlB, and RhlC enzymes involved in their synthesis. The *P. aeruginosa* quorum sensing transcriptional regulator RhlR coupled with acylhomoserine lactones endogenous produced by *P. chlororaphis* activate the transcription of the rhamnolipids structural genes forming a positive autoregulated circuit. The levels of rhamnolipids produced in the heterologous system are similar to those produced by *P. aeruginosa* PAO1.

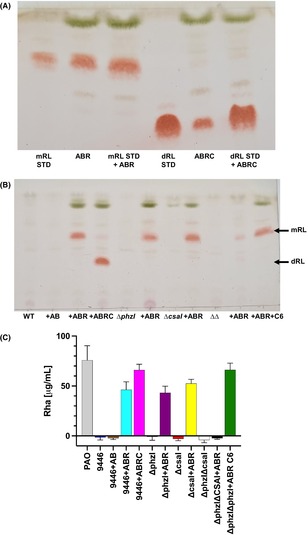

## INTRODUCTION

Biosurfactants are natural bioactive compounds (Chu et al., 2021) with surfactant properties. Thus, they have potential applications across various industrial sectors. One significant advantage they hold over chemically synthesized surfactants is their biodegradability and low toxicity (Soberón‐Chávez, 2023; Soberón‐Chávez, Hausmann, & Déziel, 2021). Rhamnolipids (RL), one of the best‐studied biosurfactants, have already entered the market and are used in cosmetics and clean‐up products, among others (Soberón‐Chávez, González‐Valdez, et al., 2021). This glycolipid is naturally produced by *Pseudomonas aeruginosa* and some *Burkholderia* species (Toribio et al., 2010), but the best producer is the former bacterium. However, the industrial use of *P. aeruginosa* for RL production is limited because this bacterium is an opportunistic pathogen that represents a significant health hazard due to its production of different virulence traits and high antibiotic resistance (Diggle & Whiteley, 2020; Gellatly & Hancock, 2013). An alternative to circumvent this problem is using heterologous hosts for RL production (Wittgens & Rosenau, 2020). In particular, *P. putida* KT2440 expressing *P. aeruginosa* genes involved in RL production serves as an efficient host (Filbig et al., 2023; Wittgens et al., 2011), and different *P. putida* KT2440‐derived strains with improved characteristics for RL production have been reported (Bator et al., 2020a, 2020b; Blesken et al., 2020).

*Pseudomonas aeruginosa* produces two forms of RL: mono‐RL, which consists of one rhamnose moiety and a dimer of fatty acids (3‐(3‐hydroxyalkanoyloxy) alkanoic acids or HAAs)—primarily β‐hydroxy decanoate molecules (C10), and di‐RL, which includes an additional rhamnose moiety. The RhlA enzyme catalyses the formation of HAAs using a CoA‐link fatty acid precursor (Abdel‐Mawgoud et al., 2014; Gutiérrez‐Gómez et al., 2019) and displays both thioesterase and acyltransferase activities (Tang et al., 2023), while RhlB uses HAAs and dTDP‐L‐rhamnose as substrates to produce mono‐RL. In turn, RhlC produces di‐RL using mono‐RL and dTDP‐L‐rhamnose as substrates (Rahim et al., 2001).

The production of RL is regulated at the transcriptional level in coordination with the production of several virulence‐associated traits by the complex regulatory network called quorum‐sensing (QS) (Williams et al., 2007). The QS‐transcriptional regulator RhlR coupled with the autoinducer butanoyl‐homoserine lactone (C4‐HSL) directly activates the transcription of the *rhlAB* operon (Croda‐García et al., 2011), and of the PA1131‐*rhlC* operon (Rahim et al., 2001). The expression of *rhlR* is positively autoregulated under some culture conditions, forming part of the *rhlAB‐R* operon (Croda‐García et al., 2011).

*Pseudomonas chlororaphis* is an innocuous soil bacterium that has been applied in agriculture as a biocontrol agent and has applications in bioremediation and other industrial fields (Anderson et al., 2018; Anderson & Kim, 2020). Most of the industrial applications of *P. chlororaphis* depend on its production of phenazines, which are naturally produced by all strains (Yu et al., 2018). These secondary metabolites, mainly phenazine‐1‐carboxylic acid (PCA) and phenazine‐1‐carboxamide (PCN) (Guo et al., 2020; Jin et al., 2016), have antibacterial and antifungal properties (Li et al., 2020). Several genetic engineering strategies have been followed to enhance *P. chlororaphis* production of these and other phenazine derivatives (Guo et al., 2020; Jin et al., 2016; Li et al., 2020; Yao et al., 2018), and some strains have been genetically modified to produce other compounds of industrial interest (Wang et al., 2018, 2021). An example of an industrial application of compounds produced by *P. chlororaphis* is the pesticide Shenqinmycin, whose primary active ingredient is PCA; it was certified since 2011 by the Chinese Agricultural Ministry (Li et al., 2020).

The *P. chlororaphis* enzymes that produce PCA are encoded by the *phzABCDEFG* operon, which is transcriptional activated by the QS‐regulator PhzR, when coupled with the acyl‐homoserine lactone (AHL) 3‐hydroxy‐hexanoyl homoserine lactone (3OH‐C6‐HSL), produced by the AHL synthase PhzI (Peng et al., 2018). In addition, strains belonging to *P. chlororaphis* subsp chlororaphis such as ATCC 9446 used in this work, have another copy of genes encoding a QS‐regulator and an AHL synthase (*csaR* and *csaI*, respectively) (Morohoshi et al., 2017). CsaR coupled with 3‐oxo‐hexanoyl homoserine lactone (3O‐C6‐HSL) produced by CsaI (Morohoshi et al., 2017, 2022) have a minor effect on phenazines production, but affects the production of cell surface properties (Zhang & Pierson, 2001). Most of the *P. chlororaphis* strains cannot produce RL, but a particular *P. chlororaphis* strain (NRRL B‐30761) can produce low amounts of mono‐RL under specific culture conditions due to its inheritance of the *rhlAB* operon through horizontal gene transfer (Gunther et al., 2005; Gunther IV et al., 2006). Some efforts to genetically modify NRRL B‐30761 strain to produce di‐RL have been reported (Solaiman et al., 2015). However, the amount produced of this biosurfactant is very low.

The genome sequence of *P. chlororaphis* strain ATCC 9446 has been reported (Moreno‐Avitia et al., 2017), and a metabolic model to maximize its production of PCN was developed, highlighting the intrinsic H_2_O_2_ flux associated with PCN production, which may generate cellular stress in phenazine overproducing strains (Bilal et al., 2017; Moreno‐Avitia et al., 2020). In this work, we use *P. chlororaphis* subsp chlororaphis ATCC 9446 as a heterologous host to produce RL. In this work, we achieve mono‐RL production by expressing the *P. aeruginosa rhlAB‐R* operon, which includes part of its 5′ untranslated region (5′ UTR). This operon is cloned into an expression plasmid with the *Escherichia coli lacZ* promoter, constitutively expressed in *P. chlororaphis*. We demonstrate that RhlR, when combined with either 3OH‐C6‐HSL or 3O‐C6‐HSL produced by *P. chlororaphis* PhzI or CsaI, activates the expression of the *rhlAB* operon encoded in this plasmid. This causes mono‐RL production and increased PCA production via the *P. chlororaphis phzABCDEG* operon. However, the chromosomally encoded PhzR, when combined with 3OH‐C6‐HSL, or CsaR when forming a complex with 3O‐C6‐HSL, are unable to stimulate the expression of the *rhlAB* operon to produce mono‐RL. The production of a particular AHL, presumably 3OH‐C6‐HSL produced by PhzI, is also increased in the strains ATCC 9446/p*rhlAB‐R* and ATCC 9446/p*rhlAB‐R‐C*. This increased level is possibly due to the induction of *phzI* expression by RhlR coupled with an AHL. These results indicate that *P. chlororaphis* subsp chlororaphis ATCC 9446, containing either the *P. aeruginosa rhlAB‐R* operon or the artificially constructed operon *rhlAB‐R‐C* (which primarily results in di‐RL synthesis), are promising biotechnological models for RL production. This production is autoregulated and can be optimized for industrial applications.

## EXPERIMENTAL PROCEDURES

### Microbiological procedures

Strains and plasmids and the oligonucleotides used in this work are shown in Supplementary information (Tables S1 and S2, respectively).

All *P. chlororaphis* strains were routinely cultured in PPGAS medium (Zhang & Miller, 1992) for 24 h at 30°C, and *Escherichia coli* strains were grown in LB medium (Miller, 1972) at 37°C.

### Construction of *P. chlororaphis* ATCC 9446 phzI and csaI mutants

The *phzI* and *csaI* genes were deleted by homologous recombination as described previously (Choi & Schweizer, 2005). To delete the *phzI* gene, primers H3Up_phzI/5phzI5Apra (Table S2) were used to amplify a 515 pb DNA fragment of the upstream sequence of this gene; the 5′ primer contained an artificial site for the restriction enzyme *Hind*III. A second fragment of 519 pb, located at the downstream region of *phzI*, was amplified with primers 3phzI3Ap/H3DwphzI (Table S2); the 3′ primer contains a sequence for the restriction enzyme *Hind*III. In addition, a 1370‐pb DNA fragment of the apramycin‐resistance cassette was amplified from pIJ773 using the primers F‐Apra/R‐Apra (Table S2). The three PCR products were purified and used as templates in a nested PCR. The PCR product was cloned into the pJET vector (Thermo Scientific), resulting in plasmid pJphzI Table S1). The 2.3‐kb fragment was excised by *Hind*III digestion from pJphzI and ligated into the suicide plasmid pEX‐Sm. The resulting plasmid was named pEX‐phzI, which was transformed into the S17‐1(λpir) *E. coli* strain, and the plasmid was introduced into *P. chlororaphis* ATCC 9446 by conjugation. The transconjugants obtained were screened for double‐crossover events, and the deletion of *phzI* was confirmed by PCR. To obtain the deletion of the *csa*I gene, we followed the same methodology, amplifying 493pb upstream of *csa*I with primers E1UpcsaI/5 csaI 5Apra (Table S2) and 537‐pb downstream of *csa*I with primers 3*csa*I 3Apra/ H3DwCsaI (Table S2) and the apramycin‐resistant cassette, resulting in the plasmids pJcsaI and pEX‐csaI.

To obtain the *phzI*, *csaI* double deletion mutant, the Δ*csaI* mutant strain was transformed with plasmid pFLP2k to excise the apramycin cassette. Transformants were selected in LB plates with kanamycin (100 μg/mL) and grown at 39°C for 16 h. Colonies that lost the resistance to apramycin but grew on kanamycin were selected. Then, the pFLP2K plasmid was cured by streaking the mutant strains on NaCl‐free LB agar plates supplemented with 10% sucrose and was confirmed by PCR. The pEX‐phzI plasmid was transferred by conjugation from *E. coli* s17λpir using the unmarked Δ*csaI* mutant as the recipient. The transconjugants generated by double recombination events were selected using antibiotic markers and were confirmed by PCR to contain the Δ*phzI* and Δ*csaI* mutations.

### Construction of the plasmid encoding the artificial operon rhlAB‐R‐C

Plasmid p*rhlAB‐R‐C* was constructed as follows: the *rhlC* gene with its ribosome binding site was amplified with PAO1 genomic DNA as a template using primers FwH3rhlC and rhlCReH3. The PCR product was digested with *Hind*III enzyme and cloned into pJGM4 plasmid in the same restriction site.

### Detection and quantification of RL

The orcinol method was used to quantified RL as rhamnose equivalents as described previously (Grosso‐Becerra et al., 2014). Briefly, a 333 μL portion of the filtered supernatant was extracted twice with 1 mL of diethyl ether. The diethyl ether was evaporated to dryness and dissolved in 1 mL of deionized water. To 100 μL of each sample, 900 μL of a solution containing 0.19% orcinol (in 53% sulfuric acid) was added. The samples were heated at 80°C in a water bath for 30 min and cooled for 15 min at room temperature, and the absorbance at 421 nm was measured. Concentrations of rhamnolipids were determined by comparing the data with those obtained with L‐rhamnose standards between 0 and 50 μg/mL. This method measures all reductive sugars present in the sample, but in this case, probably rhamnose is detected since the biosurfactant extracted from culture supernatants is the product of the activity of RhlA, RhlB, and in some instances, RhlC, which are highly specific for rhamnose.

Mono‐ and di‐RL were detected by thin layer chromatography (TLC), which was performed as follows: Each culture (5 mL) was centrifuged at 3000 *g* for 10 min, and the cell‐free supernatant was acidified to pH 2 with concentrated HCL. The RL were extracted with an equal volume of chloroform‐methanol (2:1). The organic phase of the two extractions was collected, and the solvent was evaporated to dryness. The crude extract was dissolved in 50 μL methanol, and 5 μL of the extract and 5 μL of each standard were separated by TLC on silica plates (silica gel 60; Merk) using a mixture of chloroform, methanol, and acetic acid (65:15:2). RL were visualized by spraying with an alpha‐Naphthol solution (Sigma‐Aldrich) prepared in an ethanol‐H_2_SO_4_ mixture, and heating at 90°C for 5 minutes.

### Bioassay to visualize AHLs production by *P. chlororaphis* strains

To determine the production of AHL, we used the method described previously (Grosso‐Becerra et al., 2014), which is based on the separation of AHLs by TLC, and the overlaid of the TLC plate with a thin film of agar seeded with the AHL reporter strain *C. violaceum* CV026 that produces the purple pigment violacein in response to AHLs with short *N*‐acyl side chains (Shaw et al., 1997). Synthetic hexanoyl homoserine lactone (C6‐HSL) was used as standard in this bioassay.

### RNA extraction, cDNA synthesis and qRT‐PCR assay to measure gene expression

Three RNA extractions and purifications were carried out from three independent flask fermentations for each assayed strain. Total RNA extraction was performed using hot phenol equilibrated with water, as reported (Flores et al., 2005). The resultant RNA was treated with a DNase kit (DNA‐freeTM, Ambion); determined in a NanoDrop 2000C Spectrophotometer (Thermo Scientific), and quality was determined by 260/280 nm ratio absorbance. cDNA was synthesized using RevertAidTM H First Strand cDNA Synthesis Kit (Thermo Scientific) and a mixture of specific DNA primers for *rhlA*, *phzI* and *rpoD* as housekeeping genes (Table S2). cDNA was used as the RT–PCR assay template, performed with the ABI Prism7000 Sequence Detection System and 7300 Real‐Time PCR System (Perkin Elmer/Applied Biosystems) using the MaximaR SYBR Green/ROX qPCR Master Mix (Thermo Scientific). The quantification technique to compare expression data was the $2^{-\Delta \Delta C_{T}}$ method (Livak & Schmittgen, 2001), and the results were normalized using the *rpoD* gene of *P. chlororaphis* ATCC 9446 as the internal control (housekeeping gene). The same reproducible expression level of this gene was detected in the assayed strains. For assayed genes in all strains, the transcription level of the corresponding gene in *P. chlororaphis* ATCC 9446 normalized with *rpoD* expression was considered equal to one, and it was used as a control to normalize the data reported as a relative expression level.

### Quantification of PCA

PCA concentration was determined in culture supernatants spectrophotometrically at 367 nm, which is the absorbance maximum of this compound. The concentration was calculated dividing the obtained absorbance by its molar extinction coefficient (3019) and multiplying by PCA molecular weight as described previously (Selin et al., 2010).

## RESULTS

### RhlR is indispensable for the activation of rhlAB/C genes in *P. chlororaphis* ATCC 9446 and hence for RL production

**FIGURE 1:**
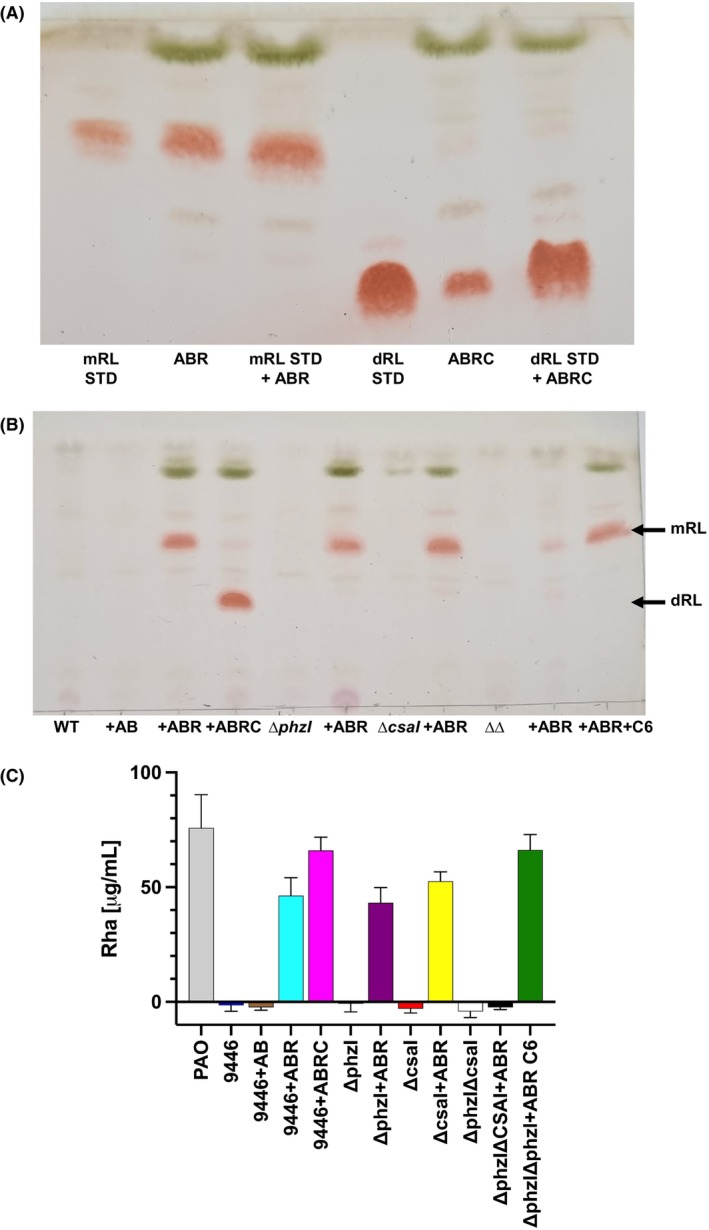
Production of RL by *P. chlororaphis* ATCC 9446 containing *P. aeruginosa rhl* genes. (A) Comparison by migration in TLC of mono‐ and di‐RL produced in *P. chlororaphis* ATCC 9446 with the same molecules produced by *P. aeruginosa* used as standards. Lanes show from left to right: mono‐RL standard (mRL STD), mono‐RL produced in *P. chlororaphis* ATCC 9446/ p*rhlAB‐R* (ABR), a 1:1 mixture of samples shown in the previous 2 lanes (mRL STD + ABR), di‐RL standard (dRL STD), di‐RL produced in *P. chlororaphis* ATCC 9446/ p*rhlAB‐R‐C* (ABRC), a 1:1 mixture of samples shown in the previous 2 lanes (dRL STD + ABRC). (B) Visualization by thin layer chromatography (TLC) of mono‐RL and di‐RL production by strains. Lanes show from left to right: ATCC 9446 (WT); ATCC 9446/p*rhlAB* (+AB); ATCC 9446/p*rhlAB‐R* (+ABR); ATCC 9446/p*rhlAB‐R‐C* (+ABRC); Δ*phzI* mutant (Δ*phzI*); Δ*phzI*/p*rhlAB‐R* (+ABR); Δ*csaI* mutant (Δ*csaI*); Δ*csaI*/p*rhlAB‐R* (+ABR); Δ*phzI*, Δ*csaI* double mutant (ΔΔ); Δ*phzI*, Δ*csaI* double mutant with plasmid p*rhlAB‐R* (+ABR); Δ*phzI*, Δ*csaI* double mutant with plasmid p*rhlAB‐R* with the addition of synthetic C6‐HSL (+ABR + C6). Mono‐ and d‐RL are shown with an arrow. (C) Quantification of RL production by the orcinol method, which measures the rhamnose equivalent present in total RL. The *P. aeruginosa* PAO1 type strain was used for comparison.

The introduction of plasmid *prhlAB‐R* to *P. chlororaphis* ATCC 9446, which encodes the positively autoregulated *P. aeruginosa* operon that catalyses the production of mono‐RL, results in the production of high titres of this biosurfactant, comparable to the amount of RL produced by PAO1 type strain (Figure 1). The production of mono‐RL by *P. chlororaphis* ATCC 9446 is entirely dependent on RhlR expression since the introduction of plasmid *prhlAB* into this strain does not result in RL production (Figure 1) or significant *rhlA* expression (Table 1).

**Table 1:** Production of phenazines (PCA and PCN) and expression of *rhlA* and *phzA* ^a^ by *P. chlororaphis* ATCC 9446 and its derivatives.

Strain	PCA (μg/mL)	Expression of *rhlA* ^a^	Expression of *phzA* ^a^
ATCC 9446	48.53 + 2.24	ND^a^	1
ATCC 9446/ p‐*rhlAB‐R*	163.52 ± 2.97	276.09 ± 37.8	38.05 ± 5.18
ATCC 9446/ p‐*rhlAB*	34.13 ± 3.71	10.57 ± 3.28	1.05 ± 0.15

^a^ The expression of *rhlA* and *phzA* was determined by qRT‐PCR. ND means not detected, but this value was considered as 1 to calculate the induction of *rhlA* expression.

Since RhlR activation of the *rhlA* promoter depends on its interaction with a short‐chain AHL, the production of mono‐RL by strain ATCC9446/*prhlAB‐R* suggests that this transcriptional regulator can form a complex with 3OH‐C6‐HSL, 3O‐C6‐HSL or both autoinducers produced by *P. chlororaphis* subsp chlororaphis ATCC 9446.

To produce di‐RL in the *P. chlororaphis* ATCC 9446 background, we constructed plasmid *prhlAB‐R‐C*, which encodes an operon expressed from the *rhlA* promoter and incorporates *rhlC* to the RhlR positively regulated loop. The expression of this plasmid results in a high titre of RL production, mainly di‐RL, which is in the same range as that produced by the PAO1 type strain (Figure 1).

Both mono‐ and di‐RL heterologously produced in *P. chlororaphis* subsp chlororaphis ATCC 9446 comigrate in a TLC plate with those produced by *P. aeruginosa* (Figure 1), showing that they have a similar composition.

### The activation of the rhlAB operon in *P. aeruginosa* by RhlR is contingent upon the AHLs produced *P. chlororaphis* ATCC 9446

To determine which of the AHL produced by *P. chlororaphis* ATCC 9446 interacts with RhlR to activate the transcription from the *rhlA* promoter, we constructed mutants with deletions of *phzI*, *csaI* or both genes.

As mentioned, PhzI produces 3OH‐C6‐HSL while CsaI catalyses the synthesis of 3O‐C6‐HSL. Our results (Figure 1) show that the production of an AHL in *P. chlororaphis* is necessary to produce RL, and that either 3OH‐C6‐HSL or 3O‐C6‐HSL can form a complex with RhlR to activate the *rhlA* promoter (Figure 1). This is apparent since Δ*phzI* or Δ*csaI* mutants containing plasmid *prhlAB‐R* can produce mono‐RL, but the double Δ*phzI*, Δ*csaI* mutant cannot produce this biosurfactant (Figure 1). Furthermore, mono‐RL production is re‐established in the Δ*phzI*, Δ*csaI* double mutant by the addition of 10 μM of synthetic C6‐HSL (Figure 1).

### RhlR, coupled with one of the AHL produced by *P. chlororaphis*, causes an increased phenazine production as well as phzA expression

Given the similar mechanism of transcription regulation of the *rhlAB* promoter by RhlR coupled with an AHL with the *P. chlororaphis* QS regulation of phenazine production, we determined whether RhlR coupled with one of the AHL produced by *P. chlororaphis* could increase their production presumably by activating the transcription of the *phzABCDEFG* promoter. We found that RhlR coupled with either of the two AHL produced by *P. chlororaphis* ATCC 9446 causes a considerable increase in the production of PCA (Tables 1 and 2). These results suggest that RhlR, coupled with either one of the AHL produced by *P. chlororaphis*, binds to the phz box upstream of the *phzA* promoter and activates the transcription of the *phzABCDEFG* operon. To test this hypothesis, we quantified *phzA* transcript by qRT‐PCR and showed that, indeed, RhlR induces the level of *phzA* transcript (Table 1).

**TABLE 2:** Quantification of PCA production by *P. chlororaphis* ATCC 9446 and its derivatives.

*P. chlororaphis s*train	PCA (μg/mL)
ATCC 9446	57.18 + 9.22
ATCC 9446 Δ*phzI*	11.90 ± 0.92
ATCC 9446 Δ*phzI*/p‐*rhlAB‐R*	142.21 ± 11.98
ATCC 9446 Δ*csaI*	40.09 ± 4.95
ATCC 9446 Δ*csaI*/p‐*rhlAB‐R*	144.02 ± 9.10
ATCC 9446 *ΔphzI*, *ΔcsaI*	11.20 ± 1.56
ATCC 9446 *ΔphzI*, *ΔcsaI*/p‐*rhlAB‐R*	14.42 ± 1.56
ATCC 9446 *ΔphzI*, *ΔcsaI*/p‐*rhlAB‐R* + C6‐HSL	148.39 ± 22.26

The concentration of PCA was used as a marker of the expression of the *phzABCDEFG* operon (Table 2). Our results with the Δ*phzI* and Δ*csaI* ATCC 9446 mutants corroborates the results previously reported (Morohoshi et al., 2022; Peng et al., 2018) that PhzR/3OH‐C6‐HSL is mainly responsible for producing phenazines, while CsaR/3O‐C6‐HSL has a minor effect on their production (Table 2).

To determine which of the AHL produced by *P. chlororaphis* was responsible for the increased phenazines production by coupling with RhlR (Table 1), we measured PCA production of the Δ*phzI* or Δ*csaI* mutants containing plasmid *prhlAB‐R*. It was apparent that RhlR coupled with either 3O‐C6‐HSL (in the Δ*phzI* mutant) or 3OH‐C6‐HSL (in the Δ*csaI* mutant) caused an increased phenazine production (Table 2). Furthermore, this increased production was also apparent in the double Δ*phzI*, Δ*csaI* mutant with plasmid *prhlAB‐R* when 10 μM of synthetic C6‐HSL was added (Table 2).

### The expression of the genes encoding autoinducer synthases PhzI and CsaI is subject to complex regulation which is affected by RhlR

**FIGURE 2:**
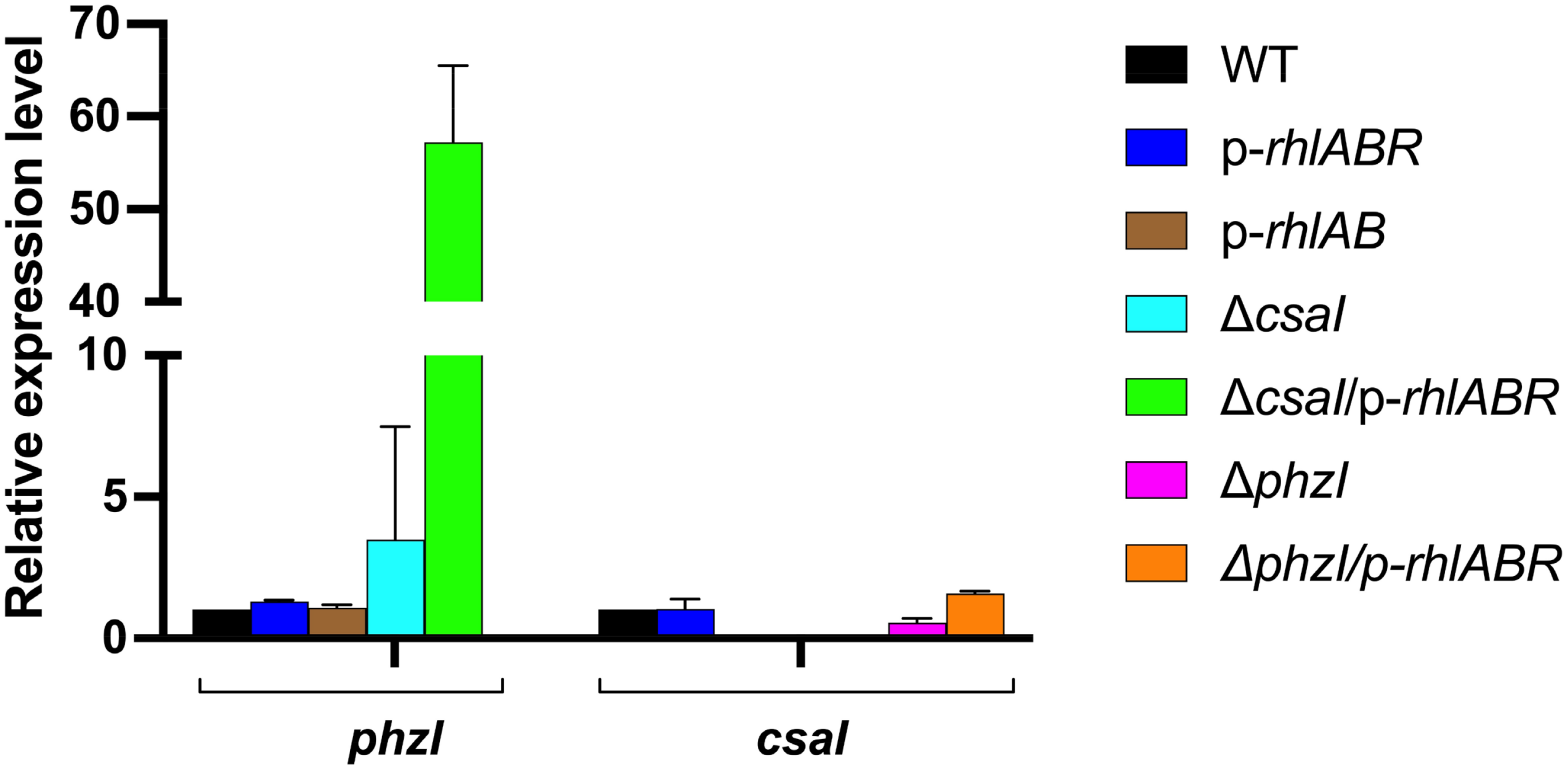
Quantification of *phzI* and *csaI* by qRT‐PCR in *P. chlororaphis* ATCC 9446 and its derived strains. The expression of *phzI* and *csaI* were measured by qRT‐PCR using the expression of *rpoD* as an internal standard. The expression of these genes in the wild‐type *P. chlororaphis* ATCC 9446 strain was considered as 1.

To determine whether *phzI* and *csaI* genes encoding the *P. chlororaphis* ATCC 9446 AHL synthases were activated by RhlR coupled with an AHL or showed a QS‐dependent regulation, we determined the expression of these genes by qRT‐PCR (Figure 2). The most evident conclusion from these experiments is that *phzI* is negatively regulated by the product of CsaI, which is 3O‐C6‐HSL (thus probably by CsaR/3O‐C6‐HSL) since its expression was increased threefold in the Δ*csaI* mutant. In the Δ*csaI* mutant, where the repression by CsaI is lifted, RhlR induced a significant increase in *phzI* expression, with an almost 60‐fold higher expression detected (Figure 2A).

**FIGURE 3:**
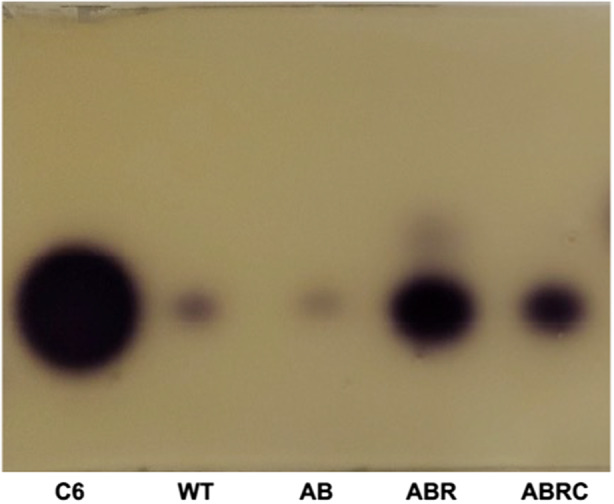
Production of AHL by *P. chlororaphis* ATCC 9446 and its derivatives. We used the bioassay based on violacein production by *C. violaceum* CV026 in the presence of short‐chain AHL. In this assay, 3O‐C6‐HSL cannot be distinguished from 3‐OH‐C6‐HSL. The results using *P. chlororaphis* ATCC 9446 (WT), ATCC 9446/*prhlAB* (AB), ATCC 9446/*prhlAB‐R* (ABR) and ATCC 9446/*prhlAB‐R‐C* (ABRC), are shown. Synthetic C6‐HSL was used as standard.

Even though the induction of *phzI* by RhlR in the wild‐type strain is not apparent (Figure 2), presumably due to the repressing effect of CsaR/3O‐C6‐HSL (Figure 2A), we detected an increased production of AHL in the presence of RhlR using the bioassay based on violacein production by *Chromobacterium violaceum* CV026 (Shaw et al., 1997) (Figure 3).

## DISCUSSION

The results presented in this work show that *P. chlororaphis* ATCC 9446 expressing the *P. aeruginosa rhlAB‐R* or *rhlAB‐R‐C* operons is a suitable system to produce mono‐ or di‐RL respectively, which has the advantage over RL production by *P. aeruginosa* of using a non‐pathogenic bacterium. In addition, this system has the advantage over the heterologous production of mono‐RL by *P. putida* KT2440 (Wittgens et al., 2011) that no inducer of *rhlAB* operon expression is needed since RhlR can form a complex with either 3O‐C6‐HSL or 3OH‐C6‐HSL naturally produced by *P. chlororaphis* ATCC 9446 (Figure 1). Furthermore, when a plasmid encoding the artificially constructed *prhlAB‐R‐C* operon was introduced *to P. chlororaphis* ATCC 9446, a comparable amount of RL was produced to PAO1, which was mainly di‐RL (Figure 1).

In addition, we show that when activating the production of RL in *P. chlororaphis* ATCC 9446, RhlR coupled with an AHL produced by this innocuous soil bacterium enhances the production of the phenazine PCA (Tables 1 and 2). The increase in phenazine formation by *P. chlororaphis* ATCC 9446 in the presence of either p*rhlAB‐R* or p*rhlAB‐R‐C* plasmids was measured spectrophotometrically (Tables 1 and 2, respectively) and is also apparent in the TLC analysis of RL (Figure 1), by the presence of the green crystal phenazine called chlororaphin (Kanner et al., 1978).

The crosstalk regulation between RhlR coupled with an AHL produced by *P. chlororaphis* ATCC 9446 and the expression of the *phzA‐G* operon might have additional biotechnological applications since phenazines produced by *P. chlororaphis* are the basis for its use as a biocontrol of several fungi (Chin‐A‐Woeng et al., 2000), and RL have also been reported to have antifungal activity (Zhao et al., 2022).

However, several RL applications might need this biosurfactant to be produced without significant amounts of other compounds. We propose that for this purpose, *P. chlororaphis* ATCC 9446 might be genetically modified to obtain mutants lacking phenazine production and redirect the carbon flow to increase RL production further.

The increased phenazine production dependent on RhlR seems to be due, at least in part, to the high increase in the expression of the *phzA* promoter of almost 40 times (Table 1). The most plausible molecular mechanism involved in the induction of the *phzABCDEFG* operon by RhlR coupled with one of the AHL produced by *P. chlororaphis* is its direct binding to the PhzR/3OH‐C6‐HSL target DNA sequence located in the *phzA* promoter region (phz box). The DNA‐binding sequence of transcriptional regulators of the LuxR family, which include PhzR, CsaR and RhlR, consists of 20 bp that contains an invariant CT separated by 12 bp from an invariant AG. The specificity of binding of each QS‐transcriptional regulator coupled with an AHL to a determined sequence depends on subtle differences in the sequences between the invariant dinucleotides, but it is not clearly understood (González‐Valdez et al., 2014); thus, the model system described here can be used for the study of this scientific question. In this respect, we show that RhlR with one of the AHL produced by *P. chlororaphis* can bind to the phz box which is located upstream of the *phzA* gene and activate its transcription. This phz box has the sequence CA ***CT*** ACAAGATCTGGT ***AG*** TT (Shah et al., 2020) while the las‐box of *rhlA* which is the target of RhlR coupled with a short‐chain AHL has the sequence TC ***CT*** GTGAAATCTGGC ***AG*** TT (nucleotides in red are shared by both binding sequences, invariant dinucleotides are written in bold and italic characters). However, PhzR/3OH‐C6‐HSL only recognizes the *phzA* phz box, and not the *rhlA* las‐box. This difference in the specificity of both QS‐transcriptional regulators shows that contact with different nucleotides is essential for each of them and that there is no straightforward correlation to conclude which are the important nucleotides for their interaction.

We also detected that RhlR causes an increase in AHL production (Figure 3) even though no increase in the expression of *phzI* or *csaI* was detected in the wild‐type ATCC 9446 carrying plasmid *prhlAB‐R* (Figure 2). This apparent contradiction might be due to an increased expression of *phzI* caused by RhlR coupled with AHL at a different moment along the growth curve than the 24‐h point when we extracted the RNA to perform the qRT‐PCR experiments. This possibility is sustained by the high *phzI* induction caused by RhlR in the Δ*csaI* mutant (Figure 2).

The production of high levels of both mono‐ and di‐RL by *P. chlororaphis* ATCC 9446 expressing *P. aeruginosa* genes are encouraging results for the potential use of this bacterium as a heterologous host, but this work represents just a proof of concept. Several fields of research using this innocuous soil bacteria for RL production should be followed to determine whether it is feasible to exploit it at the commercial level and whether some of the challenges that the expansion of the RL market face (Dittmann et al., 2023) can be solved using this model system. For example, it is important to purify the RL produced by *P. chlororaphis* ATCC 9446 with plasmid *prhlAB‐R* or *prhlAB‐R*‐C and characterize their structure to see whether they contain the same fatty acid congeners as the ones produced in *P. aeruginosa* and to directly confirm that L‐rhamnose is the sugar present in these glycolipids; to optimize the culture medium composition and fermentation conditions for this model bacterium; and to follow different metabolic engineering strategies to improve RL productivity using cheap substrates. All these strategies have been followed with *P. putida* KT2440 expressing the *P. aeruginosa rhlAB* operon (Filbig et al., 2023), and a robust system for RL production has been developed. However, the model we describe here might become an alternative for the industrial production of RL with some advantages, such as its autoinduction capacity.

In conclusion, the results presented here show the great biotechnological potential of the heterologous production of RL using *P. chlororaphis* as a host and highlight possible strategies to further increase RL production using this innocuous soil bacterium.

## AUTHOR CONTRIBUTIONS

**Abigail González‐Valdez:** Conceptualization (equal); data curation (equal); investigation (equal); methodology (equal); project administration (equal); writing – review and editing (equal). **Adelfo Escalante:** Formal analysis (equal); investigation (equal); methodology (equal); writing – review and editing (equal). **Gloria Soberón‐Chávez:** Conceptualization (lead); formal analysis (equal); funding acquisition (lead); resources (lead); supervision (lead); validation (lead); writing – original draft (lead); writing – review and editing (equal).

## CONFLICT OF INTEREST STATEMENT

The authors declare that they do not have any conflict of interest.

## Supporting information


Table S1.
Table S2.
