# Supplementary material for: Heterologous production of rhamnolipids in Pseudomonas chlororaphis subsp chlororaphis ATCC 9446 based on the endogenous production of N‐acyl‐homoserine lactones
Source: Microb Biotechnol. 2023 Dec 2;17(1):e14377. doi: 10.1111/1751-7915.14377 (PMC10832566; doi:10.1111/1751-7915.14377)
Supplement: Supplementary file 1 — Table S1. Table S2. [file MBT2-17-e14377-s001.docx]

**Supplementary information**

Title: Heterologous production of rhamnolipids in *Pseudomonas chlororaphis* subsp chlororaphis ATCC 9446 based on the *Pseudomonas aeruginosa* RhlR-dependent expression of the *rhlAB* operon and *rhlC* gene, and the endogenous production of *N*-acyl-homoserine lactones.

Authors: Abigail González-Valdez^1^, Adelfo Escalante^2^, Marco Antonio Morales-Escalante^2^, Gloria Soberón-Chávez*^1^.

Affiliations: ^1^Departamento de Biología Molecular y Biotecnología, Instituto de Investigaciones Biomédicas, Universidad Nacional Autónoma de México, Ciudad Universitaria, Apdo. Postal 70228, C. P. 04510, CDMX, México.

^2^Departamento de Ingeniería celular y Biocatálisis, Instituto de Biotecnología, Universidad Nacional Autónoma de México. Avenida Universidad 2001. Chamilpa. C. P. 62210. Cuernavaca, Morelos. México.

*Corresponding author: Gloria Soberón-Chávez. Departamento de Biología Molecular y Biotecnología, Instituto de Investigaciones Biomédicas, Universidad Nacional Autónoma d México, Ciudad Universitaria, Apdo. Postal 70228, C. P. 04510, CDMX, México; 52-55-56229201; [gloria@iibiomedicas.unam.mx](mailto:gloria@iibiomedicas.unam.mx)

**Table S1-Strains and plasmids used in this work**

| **Strains** | | |
| --- | --- | --- |
| **Name** | **Relevant genotype/phenotype** | **Reference** |
| ***Escherichia coli*** | | |
| DH5α | *lacZ*ΔM15 Δ(*lacZYA-argF*)U169 *recA1 endA1 hsdR17* [rKmK+] *supE44 thi-1 gyrA relA1* | Lab Stock |
| S17-1λ pir | *(F -) RP4-2-Tc::Mu aphA::Tn7recApir lysogen* | (Simon *et al.*, 1983) |
| ***Pseudomonas chlororaphis*** | | |
| ATCC9446 | Wild type strain used in this work.  Cb^R^, Sm^R^. | ATCC Collection |
| *ΔphzI* | ATCC9446 ΔphzI::FRT-aac(3)IV-FRT (*Ap*^R^) | This work |
| *ΔCsaI* | ATCC9446 ΔcsaI::FRT-aac(3)IV-FRT (*Ap*^R^) | This work |
| *Double I* | ATCC9446 ΔcsaI::FRT ΔphzI::FRT-aac(3)IV-FRT (*Ap*^R^) | This work |
| ***Chromobacterium violaceum*** | | |
| *CV026* | AHL biosensor, ATCC 31532 derivative, cviI::Tn5xylE, (Km^R^) | (Shaw *et al.*, 1997) |
| **Plasmids** | | |
| **Name** | **Relevant genotype/phenotype** | **Reference** |
| pJET1.2 | pJET1.2 blunt cloning vector (Cb^R^) | Thermo  Scientific |
| pUC4K | Source of kanamicyn resistant cassette (Cb^R^Km^R^) | (Taylor and Rose, 1988) |
| pEX-Sm | Sm^R^ ; oriT , sacB , gene replacement | (García-Reyes *et al.*, 2021) |
| pIJ773 | Plasmid containing aac(3)IV gene apramycin. *(Cb^R^ , Ap*^R^) | (Gust *et al.*, 2003) |
| pFLP2 | *sacB,* λ *cI*857 repressor, *FLP recombinase, bla* (Cb^R^) | (Hoang *et al.*, 1998) |
| pJMG4-*rhlAB-R* | pUCP24 derived plasmid with a 2.7 kb fragment containing PAO1 *(Cb^R^* ) | (Grosso-Becerra *et al.*, 2016) |
| pUCP20-*rhlABR* | pUCP20 derived plasmid with a 2.7 kb fragment containing PAO1 *(Cb^R^* ) | (Gutiérrez-Gómez *et al.*, 2018) |
| pJphzI | Ligation of a 2.3-kb fragment including 5’-UTR and 3’-UTR from gene *phzI* flanking for HindIII *FRT -aac (3) IV-FRT* -HindIII *(Cb^R^ , Ap*^R^) | This work |
| pJcsaI | Ligation of a 2.4-kb fragment including 5’-UTR and 3’-UTR from gene *phzI* flanking for EcoRI *FRT -aac (3) IV-FRT* -HindIII *(Cb^R^ , Ap*^R^) | This work |
| pEX-phzI | Subcloning of a 2.3 Kb of *phzI::FRT -aac (3) IV-FRT at Hind*III *site of* pEX-Sm *(sm^R^ , Ap*^R^) | This work |
| pEX-csaI | Subcloning of a 2.4 Kb of *csaI::FRT -aac (3) IV-FRT between EcoRI and Hind*III *sites of* pEX-Sm *(sm^R^ , Ap*^R^) | This work |
| pABRk | Constructed by ligation the 1.2-kb kanamycin cassette cloned at scaI site in pUCP20-rhlABR plasmid *(km^R^*) | This work |
| pAB-R-C | Constructed by ligation the 1.0-kb *rhlC* gene cloned at *Hind*III site in pJGM4-rhlABR plasmid (*Gm^R^*) | This work |
| pFLP2k | Constructed by ligation the 1.2-kb kanamycin cassette cloned at *Sca*I site in pFLP2 plasmid (*km^R^*) | This work |

**Antibiotic abbreviations**: Sm, stretomycin; Cb, carbenicilin; Apra, apramycin. Km. Kanamicyn.

**Table S2-Oligonucleotides used in this work**

| **Name** | **Sequence (5´- 3´)** | **Source** |
| --- | --- | --- |
| H3Up_phzI | CCAAGCTTAAAGCGCTTGACGCCTTAC | This work |
| 5phzI5Apra | GTCGACGGATCCCCGGAATACATTACTGAAGTTTTCCTTAGGG | This work |
| 3phzI3Ap | GAAGCAGCTCCAGCCTACATGATACAGGCCGAGTCGTCC | This work |
| H3DwphzI | CCAAGCTTTCGTCCCCCATTTTGTGG | This work |
| E1UpcsaI | GAATTCCACGGAAATGGTGGTCTGGA | This work |
| 5 csaI 5Apra | GTCGACGGATCCCCGGAATAGATCATGGGGCGATATCCTT | This work |
| 3 csaI 3Apra | GAAGCAGCTCCAGCCTACAGCAACCCACCACCAACGT | This work |
| H3DwCsaI | AAGCTTCGATGGTGCTGTTCTTCGTC | This work |
| rhlCReH3 | GCGTTTAAGCTTCTAGGCCTTGGCCTTGCCGG | This work |
| FwH3rhlC | CGGGCTAAGCTTGGCCTGGCAACTTCGACCTA | This work |
| F-Apra | TATTCCGGGGATCCGTCGAC | (Gust *et al.*, 2003) |
| R-Apra | TGTAGGCTGGAGCTGCTTC | (Gust *et al.*, 2003) |
| F-kan | AAAGCCACGTTGTGTCTCAAAATC | (Lesic and Rahme, 2008) |
| R-kan | GCGCTGAGGTCTGCCTCGTGAAGA | (Lesic and Rahme, 2008) |
| **Oligonucleotides used for RT-qPCR** | | |
| **Target gene** | **Sequence (5’-3’)** | **Source** |
| rpoDPa | GGC AGT GGA AAC CGA CAT TG | This work |
| rpoDPa | TTT CGA TTT CGC CTT CAC GT | This work |
| rhlAa | TGA TCA CCA AGG ACG ACG AG | This work |
| rhlAb | CAG CGT GGA GAT ACC GCC | This work |
| phzIa | GCA CCA TGG AGC GCT ATT ACA | This work |
| phzIb | CGA GAG TTT GAT GGC GAG GA | This work |
| csaIa | TGA TTT CAC GGC ATG AAA GC | This work |
| csaIb | GGG CAG TCG CCA ACC C | This work |
| phzAf | TCG TCC AGT TCG AAA GAA TGG | This work |
| phzAr | CAC TTC TGG GTG GAA AGC GA | This work |

**References**

García-Reyes, S., Cocotl-Yañez, M., Soto-Aceves, M.P., González-Valdez, A., Servín-González, L., and Soberón-Chávez, G. (2021) PqsR-independent quorum-sensing response of *Pseudomonas aeruginosa* ATCC 9027 outlier-strain reveals new insights on the PqsE effect on RhlR activity. *Mol Microbiol* 116: 1113–1123.

Grosso-Becerra, M.-V., González-Valdez, A., Granados-Martínez, M.-J., Morales, E., Servín-González, L., Méndez, J.-L., et al. (2016) *Pseudomonas aeruginosa* ATCC 9027 is a non-virulent strain suitable for mono-rhamnolipids production. *Appl Microbiol Biotechnol* 100: 9995–10004.

Gust, B., Challis, G.L., Fowler, K., Kieser, T., and Chater, K.F. (2003) PCR-targeted *Streptomyces* gene replacement identifies a protein domain needed for biosynthesis of the sesquiterpene soil odor geosmin. *Proc. Natl. Acad. Sci. U.S.A.* 100: 1541–1546.

Gutiérrez-Gómez, U., Soto-Aceves, M.P., Servín-González, L., and Soberón-Chávez, G. (2018) Overproduction of rhamnolipids in *Pseudomonas aeruginosa* PA14 by redirection of the carbon flux from polyhydroxyalkanoate synthesis and overexpression of the rhlAB-R operon. *Biotechnol Lett* 40: 1561–1566.

Hoang, T.T., Karkhoff-Schweizer, R.R., Kutchma, A.J., and Schweizer, H.P. (1998) A broad-host-range Flp-FRT recombination system for site-specific excision of chromosomally-located DNA sequences: application for isolation of unmarked *Pseudomonas aeruginosa* mutants. *Gene* 212: 77–86.

Lesic, B. and Rahme, L.G. (2008) Use of the lambda Red recombinase system to rapidly generate mutants in Pseudomonas aeruginosa. *BMC Mol Biol* 9: 20.

Shaw, P.D., Ping, G., Daly, S.L., Cha, C., Cronan, J.E., Rinehart, K.L., and Farrand, S.K. (1997) Detecting and characterizing N-acyl-homoserine lactone signal molecules by thin-layer chromatography. *Proc. Natl. Acad. Sci. U.S.A.* 94: 6036–6041.

Simon, R., Priefer, U., and Pühler, A. (1983) A broad host range mobilization system for in vivo genetic engineering: transposon mutagenesis in gram negative bacteria. *Nat Biotechnol* 1: 784–791.

Taylor, L.A. and Rose, R.E. (1988) A correction in the nucleotide sequence of the Tn903 kanamycin resistance determinant in pUC4K. *Nucleic Acids Res* 16: 358.
